# 

**Published:** 1869-02

**Authors:** 


VOL. V. *January & February*, 1869. No. 4.

THE

### IOWA MEDICAL JOURNAL,

EDITED BY

J. C. HUGHES, M. D.,

*Professor of the Institutes, and Practice of Surgery and Surgical Clinics in the Medical Department Iowa State University; Surgeon-in-Charge of the College Infirmary and Eye and Ear Institute.*

SMITHSONIAN INSTITUTION

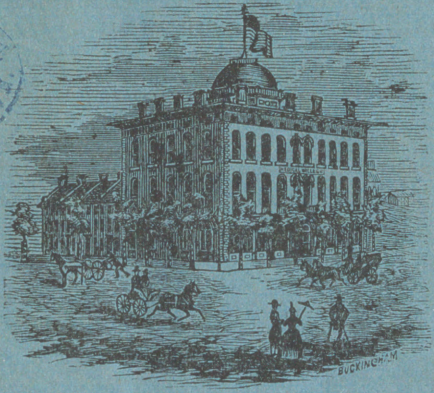

KEOKUK, IOWA.

Published every alternate Month.

Price, Two Dollars a Year, in advance.

Editors and Publishers Please Exchange.

Address all Communications, Periodicals or Books to the Editor.

*KEOKUK, IOWA:*
PRINTED AT THE GATE CITY BOOK AND JOB ROOMS.
1869.

Letters requiring an answer, must contain a Stamp to insure attention.

